# *Cyclin L1 (CCNL1)* gene alterations in human head and neck squamous cell carcinoma

**DOI:** 10.1038/sj.bjc.6603036

**Published:** 2006-04-04

**Authors:** D Muller, R Millon, S Théobald, T Hussenet, B Wasylyk, S du Manoir, J Abecassis

**Affiliations:** 1 Laboratoire de Biologie Tumorale, Centre de lutte contre le cancer Paul Strauss, 3 rue de la Porte de l'Hôpital, 67065 Strasbourg Cedex, France; 2 Service de statistique et d'épidémiologie, Centre de lutte contre le cancer Paul Strauss, 3 rue de la Porte de l'Hôpital, 67065 Strasbourg Cedex, France; 3 Institut de Génétique et de Biologie Moléculaire et Cellulaire, CNRS/INSERM/Collège de France, 1 rue Laurent Fries, BP10142, Illkirch Cedex 67404, CU de Strasbourg, France

**Keywords:** head and neck, squamous cell carcinoma, cyclin L1, CCNL1, chromosome 3q, HNSCC

## Abstract

We evaluated the expression and amplification of *cyclin L1* (*CCNL1)* gene, a potential oncogene localised in the commonly amplified 3q25–28 region, in human head and neck squamous cell carcinomas (HNSCCs). Overexpression was observed in 55 out of 96 cases (57%) and amplification in nine out of 35 tumours (26%) with no relationships to the clinico-pathological parameters. The Cyclin L1 antibody we developed labels nuclear speckles in tumour cells compatible with a role for CCNL1 in RNA splicing.

Definition of the genetic changes associated with head and neck squamous cell carcinoma (HNSCC), in particular by comparative genomic hybridisation (CGH), pinpointed gain of the 3q chromosome as one of the most frequent abnormalities (Bockmuhl *et al*, 2000). We demonstrated that the 3q25–28 region is a consistent site of gain in early HNSCC (Redon *et al*, 2001), and suggested that the gain is associated with shortened disease survival (Redon *et al*, 2001). This association was confirmed in other larger studies underlying the importance of this region in the development of HNSCC (Bockmuhl *et al*, 2000; Ashman *et al*, 2003, and publications therein). Several putative oncogenes from the 3q25–28 region have been proposed to be potential targets of amplification, including *PIK3CA* (Estilo *et al*, 2003), *SCCRO* (Estilo *et al*, 2003), *p63* (Thurfjell *et al*, 2005), and *cyclin L1 (CCNL1),* which we showed by microarray-based CGH to be amplified in an HNSSC cell line (Redon *et al*, 2002). The main *α* isoform of Cyclin L1 contains an RS domain (Ser–Arg rich proteins) that may confer splicing activity to cyclin L1 (Dickinson *et al*, 2002). *CCNL1* is an immediate early gene induced by several growth factors (Berke *et al*, 2001) that may regulate G0–G1 cell-cycle progression (Redon *et al*, 2002 and publications herein).

In the present study, we measured *CCNL1* gene expression in 96 human HNSCCs by real-time quantitative PCR. *CCNL1* gene copy number was evaluated to examine the relationship between RNA expression and DNA copy number alterations. The relationship between *CCNL1* gene alterations and pathological features as well as clinical outcome is reported. Finally, the localisation of the cyclin L1 protein was determined by immunohistochemistry on normal and neoplastic head and neck tissues.

## MATERIALS AND METHODS

### Patients and samples

A total of 96 HNSCC samples were selected from consenting patients who underwent surgery as first treatment, without previous radiation or chemotherapy. Matched normal mucosa samples were obtained for 82 cases by resection at least 5 cm from the tumour. They were immediately frozen and stored in liquid nitrogen. Clinico-pathological features are summarised in Table 1 using the UICC TNM system. The mean clinical follow-up was 62 months (range 0.5–199).

### *Cyclin L1* gene expression and copy number

Total RNA isolation and cDNA preparation were performed as described previously (Redon *et al*, 2001). Genomic DNA was isolated by phenol/chloroform extraction. *CCNL1* gene amplification and expression were assessed by quantitative PCR performed with the LightCycler system, using LC Fast start DNA master SYBR green I mixture, and version 3.0 software (Roche Diagnostics, Meylan, France).

The specific oligonucleotide primers, designed by Primer3 software, were: (a) for gene expression analysis: *CCNL1,* 5′-ACTCCAAGCCCCCTGATCCT-3′ and 5′-TGGCAACGGAATCTGAAGTG-3′, which amplify the *α* and *β* isoforms; the ubiquitous gene *RPLP0,* 5′-GAAGGCTGTGGTGCTGATGG-3′ and 5′-CCGGATATGAGGCAGCAGTT-3′; (b) for gene amplification analysis: *CCNL1,* 5′-TAGGCGGAGTCGATCTGGAA-3′ and 5′-CCATGGTGCTTGCTTTTATGG-3′; and two genes located on respectively chromosome 15q15–21 and 11p15, CAPN3 (calpain3/p94), 5′-GCTGGTAGGAGACCCCCAAG-3′ and 5′-CCACAGATGCGCTAATGACG-3′, HBB (beta-globin), 5′-GAAGAGCCAAGGACAGGT-3′ and 5′-TGGTGTCTGTTTGAGGAAGC-3′.

In order to obtain for each gene results in ng for expression and copy number, respectively, calibration curves were constructed using pools of cDNAs from 10 normal head and neck tissue samples and DNA from peripheral blood samples from 10 healthy individuals. *CCNL1* gene expression was evaluated for 96 tumours and 82 corresponding normal tissues using the mean value from three independent experiments. The relative *CCNL1* gene expression level was calculated by successive normalisation to the RPLP0 (Ribosomal phospho-protein P0) internal control and then to the mean expression of all the normal tissue samples. A relative value ⩾1.7 was considered to represent overexpression. *Cyclin L1* copy number changes were analysed for 35 tumours and 14 normal samples by independent duplicate PCR reactions with two DNA inputs, 6 and 2 ng. Copy number values for a tumour sample were calculated in relative units adjusted to the mean values of CAPN3 and HBB internal controls. The results were expressed as fold differences in target gene copy number in tumours relative to normal samples. A relative value ⩾1.5 was considered to represent amplification.

### Cyclin L1 protein expression

Cyclin L1 protein expression was assessed by immunohistochemistry, using a mouse monoclonal antibody raised against the peptide SKHHGGRSGHCRHRR. This sequence is found only at the C-terminal of the isoform *α*. For retrieval, 3 *μ*m de-waxed sections were pressure-cooked for 3 min in 0.1 M citrate buffer pH 6.0. They were incubated overnight at 4°C with a 1/1000 dilution of the primary antibody, and revealed by the avidin-biotin peroxydase complex method (DAKO labelled streptavidin-biotin LSAB). Negative controls lacked the primary antibody.

### Statistics

Associations with clinico-pathological features and *CCNL1* expression or amplification were analysed at the 5% significance level using the ANOVA or Kruskal Wallis tests where appropriate. Survival was analysed by the Kaplan–Meier method and the log-rank test with MedCalc software (MedCalc, Belgium).

## RESULTS

**Figure 1:**
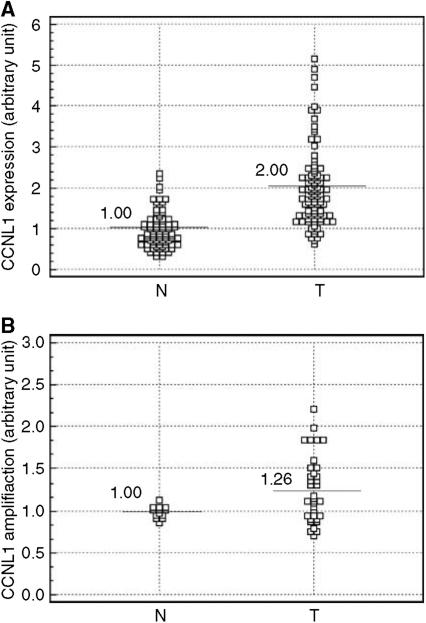
*CyclinL1* gene alterations assessed by quantitative real-time PCR. (**A**) Relative *cyclin L1* gene expression level in 96 head and neck tumours (T) and 82 normal tissues (N); *T*-test, *P*<0.0001, mean value for N: 1.00 by construction (95% CI=0.9–1.09), for T: 2.01 (95% CI=1.8–2.2), (**B**) Relative *cyclin L1* gene copy level in 14 normal (N) and 35 tumour tissues (T); *T*-test, *P*=0.02, mean value for N: 1.00 by construction (95% CI=0.95–1.05), for T: 1.26 (95% CI=1.12–1.39).

Real-time quantitative PCR analysis of 96 HNSCCs and 82 of their counterpart normal tissues showed that *Cyclin L1* gene expression levels were significantly different between tumours and normal tissue samples (*T*-test; *P*<0.0001) (Figure 1A). Overexpression was found in 55 tumours (57%). Expression levels in tumours were not significantly associated with their clinico-pathological features (tumour site, size or differentiation, lymph node involvement, Table 1).

*CCNL1* gene amplification, evaluated in 35 HNSCCs, was observed in 26% of the tumours and does not exceed three-fold (relative to the mean of 14 normal tissue samples) (Figure 1B). Overexpression was observed in almost all tumours with amplification (seven out of nine cases with amplification), while lack of overexpression in tumours without amplification was observed in 65% (17 out of 26) of the cases, suggesting correlation between *CCNL1* overexpression and amplification (Table 2, Fisher's test *P*=0.049). Amplification was not statistically related to the clinico-pathological features of the tumours (not shown).

Disease progression was observed for 60 patients. No statistically significant relationship was observed between either *CCNL1* overexpression or amplification and crude survival or relapse-free survival of the patients, even for tumours with high expression levels (highest quartile, data not shown). Gene amplification evaluation was mainly performed on tumours of pharyngeal origin (86%). For the tumours with concomitant overexpression and amplification, no association with either histo-clinical features or survival outcome was observed.

**Figure 2:**
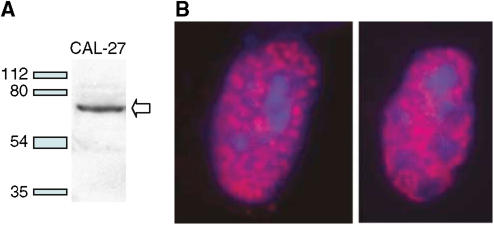
Validation of anti Cyclin L1 alpha mouse monoclonal antibody. (**A**) Western blot validation. The monoclonal antibody was validated with a Cal-27 HNSCC cell line protein extract. It detects a protein (indicated by the arrow) of size between 54 and 80 kDa, compatible with predicted size of CCNL1 alpha (526 aa, around 60 kDa). Molecular marker sizes are indicated on the left. (**B**) Immunocytofluorescence validation. The CCNL1 alpha antibody (red colour) labels nuclear speckles of the two transfected COS cells presented. No cytoplasmic staining was observed. Blue colour corresponds to DAPI staining of nuclei. This pattern is identical to a previously published localisation using an anti-FLAG labelling on transfected COS cells (Berke *et al*, 2001).

**Figure 3:**
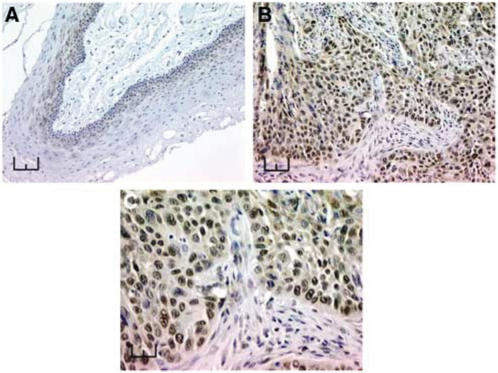
Immunohistochemical detection of cyclin L1 protein in normal and neoplastic head and neck tissues from HNSCC patients. (**A**) Slight positive staining of parabasal cells (arrow) of normal epithelium from the upper aero-digestive tract (Bar represents 200 *μ*m). (**B**) Strong nuclear staining of tumour cells in a moderately differentiated head and neck tumour tract (bar represents 50 *μ*m); (**C**) enlargement of image B, positive staining of speckles in the nuclei tract (bar represents 25 *μ*m).

We developed a monoclonal antibody against the long form of the protein (*α* isoform) and used it for Western blotting (Figure 2A). The nuclear staining (Figure 2B) observed by immunocytofluorescence is similar to that observed in COS cells transfected with flagged CCNL1 and revealed by an anti-FLAG antibody (Berke *et al*, 2001). In tumours, Cyclin L1 staining is mostly restricted to the nuclei of the epithelial cells with discrete additional cytoplasmic staining in some cells (Figure 3). No staining was observed in stromal cells. Heterogeneous staining was observed throughout the tumour sections and between different tumours. Normal epithelium displays weak nuclear staining of the parabasal cells (arrow).

## DISCUSSION

In this large series of HNSCCs, we observed a high prevalence of *CCNL1* gene overexpression, occurring in more than half of the tumours. *CCNL1* gene copy number was increased in 26% of the cases, in accordance with the 34% of gains reported by FISH analysis with a *CCNL1* gene probe (Sticht *et al*, 2005). Furthermore, we demonstrated gene overexpression in most HNSCCs with amplification of the *CCNL1* gene, supporting the hypothesis that its expression level partly follows a gene dosage effect. However, *CCNL1* overexpression was also detected in 34% (nine out of 26) of tumours without *CCNL1* gene amplification, showing that other transcriptionnal regulation factors are involved. Similar discrepancy between overexpression and amplification has been reported for other oncogenes. For example, overexpression of cyclin D1 in oral squamous cell carcinomas is twice as frequent as *CCND1* amplification (Miyamoto *et al*, 2003).

A recent study, comparing DNA copy number to gene expression levels over large chromosomal regions in HNSCCs, concludes that chromosomal alterations affect the expression of many genes (Masayesva *et al*, 2004). The discrimination between genes that are important for tumour evolution from bystander ones within regions of copy number changes remains complex. Only some of these overexpressed genes may be important for tumorigenesis, and identifying them replies on several clues such as gene amplification/expression correlations, functional characterisation or association with clinical features. Multiple genes within the commonly gained 3q region appear to be relevant for head and neck cancer progression. The gain of chromosomal region 3q25–28 is associated with aggressive clinical behavior (Bockmuhl *et al*, 2000; Singh *et al*, 2002; Ashman *et al*, 2003). Overexpression of several genes at 3q26 have prognostic value, including *SCCRO* in oral tongue (Estilo *et al*, 2003) and *ZASC1* in oesophageal (Imoto *et al*, 2003) tumours. Recently, high levels of *CCNL1* gene amplification, assessed by FISH (>8 signals/cell) have been found in 3% of a large HNSCC series and were shown to be associated with shorter survival (Sticht *et al*, 2005). In our series, high-level amplification was not found, and neither *CCNL1* gene amplification nor overexpression was associated with unfavourable tumour phenotype. Difference concerning amplification between both studies may be due to sampling differences since our population is mainly composed of tumours derived from pharynx (86 %) while Sticht *et al* investigated mostly oral cavity tumours.

Inappropriate expression of multiple genes at the 3q25–28 chromosomal locus, including *CCNL1*, could contribute to disease progression, providing growth advantage by additive effects in different cellular pathways. *CCNL1* (isoform *α*) could be a regulator of the G0 to G1 cell-cycle transition. Analysis of the relationships with cell cycle regulators specially relevant in head and neck cancers, such as Cyclin D1 or growth factors, would be of interest. *CCNL1* may have also a role in transcription or RNA splicing, since it colocalises within nuclear speckles with splicing factors SC-35 and 9G8 in cells transfected by *cyclin L1* isoform *α* (Yang *et al*, 2004). Interestingly, cyclin L1 was also observed in the nuclei of malignant cells of head and neck tumours, by histological immunodetection using our antibody. CCNL1 overexpression may modify the splicing pattern of specific genes, in particular genes involved in apoptosis for which splicing variants have been shown to have agonist or antagonist effects (Schwerk and Schulze-Osthoff, 2005). Altogether, these findings suggest that cyclin L1 might have a role in the RNA processing complex and could participate to tumour progression of HNSCC. It thus remains of interest to investigate further the physiological functions of cyclin L1 and its link to head and neck carcinogenesis.

## Figures and Tables

**Table 1:** Relation between *Cyclin L1* gene expression and clinico-pathological features of tumours

	**Number of cases**	**Overexpression Number of cases (%)**	***P*-value** *
*Localisation*			
All	96	55 (57%)	
Hypopharynx	64	38 (59%)	
Oropharynx	20	13 (65%)	0.245
Oral cavity	12	4 (33%)	
			
Tumour size			
T1	12	7 (58%)	
T2	49	33 (67%)	0.065
T34	35	15 (43%)	
			
*Lymph node involvement*			
N0	44	22 (50%)	0.59
N+	52	33 (63%)	
			
*Tumour differentiation*			
Well	30	13 (43%)	0.34
Moderately	34	22 (65%)	
Poorly to undifferentiated	32	20 (62%)	

* ANOVA test on relative expression values.

**Table 2:** Relationship between *cyclin L1* gene amplification and expression in head and neck SCC

	**Not amplified**	**Amplified**	**Total**
No overexpression	17	2	19
Overexpression	9	7	16
Total	26	9	35

Fisher's test, *P*=0.0497.

SCC, squamous cell carcinoma.
